# Effects of 4‐Week Treadmill Running at Different Intensities on Exercise‐Induced Hypoalgesia and Endogenous Pain Modulation in Healthy Individuals

**DOI:** 10.1155/prm/8255039

**Published:** 2026-02-26

**Authors:** Zihan Xu, Nan An, Shuang Xu, Ruyun Wang, Yue Li

**Affiliations:** ^1^ School of Sport Medicine and Rehabilitation, Beijing Sport University, Beijing, China, bsu.edu.cn; ^2^ Division of Surgery and Interventional Science, University College London, London, UK, ucl.ac.uk; ^3^ Special Education College, Beijing Union University, Beijing, China, buu.edu.cn

**Keywords:** endogenous pain modulation, exercise-induced hypoalgesia, moderate-intensity exercise, pain, treadmill running

## Abstract

**Objectives:**

We aimed to investigate changes in pain perception, acute exercise‐induced hypoalgesia (EIH), and endogenous pain modulation responses following 4‐week treadmill running exercises of different intensities in healthy individuals.

**Methods:**

Fifty‐six healthy individuals were included in this study. All participants were randomly assigned to a control group and three experimental groups (treadmill running at high intensity [TRH], treadmill running at moderate intensity [TRM], and treadmill running at low intensity [TRL]). All participants performed 12 treadmill running sessions within 4 weeks at different intensities based on their target heart rate (THR). A running assessment was administered 1 week before running sessions. The magnitudes of EIH, conditioned pain modulation (CPM), and temporal summation (TS) responses following regular treadmill running were assessed. Pressure pain thresholds (PPTs) or mechanical pain thresholds (MPTs) were also determined following regular treadmill running.

**Results:**

All groups exhibited an EIH effect (*p* < 0.001, *F* = 9.424) with an increase in PPT and MPT during the running sessions (*p* = 0.004 and *F* = 2.084), and the TRM and TRL groups were significantly higher than the TRH group (*p* < 0.001). The CPM of the TRM and TRL groups significantly increased (*p* < 0.001), and the TS of the TRM significantly decreased (*p* < 0.001). Correlation analysis showed that the acute EIH‐A (*r* = 0.724, *p* < 0.001), EIH‐L (*r* = 0.726, *p* < 0.001), and EIH‐M (*r* = 0.347, *p* = 0.009) were positively correlated with the CPM, while EIH‐A (*r* = −0.529, *p* < 0.001) and EIH‐L (*r* = −0.544, *p* < 0.001) were negatively correlated with the TS.

**Conclusion:**

A 4‐week low‐to‐moderate intensity treadmill running improved acute EIH response by enhancing endogenous pain modulation in healthy individuals. Future studies should consider sex, behavior, and physiological factors to provide a comprehensive understanding of the changes in EIH following regular exercises.

**Trial Registration:**

ClinicalTrials.gov identifier: ChiCTR2300074367

## 1. Introduction

Acute reduction of pain perception in the body following a single bout of exercise, commonly called exercise‐induced hypoalgesia (EIH), has been widely confirmed in healthy individuals and pain patients with fibromyalgia, chronic low back pain, and knee osteoarthritis [1]. Usually, both the global aerobic (e.g., treadmill running and cycling) [2] and localized resistance exercise (e.g., isometric wall squats) [3] with a certain intensity and duration can temporarily increase various pain thresholds (pressure pain thresholds [PPTs] or mechanical pain thresholds [MPTs]) and enhance emotional well‐being [4]. EIH can also manifest as both local effects (at the exercised muscles) and global effects (at remote, nonexercised areas), with local effects typically being more pronounced. However, the EIH response may be weakened (absence of hypoalgesia or hyperalgesia) in older adults [5] or patients with painful conditions such as fibromyalgia, knee osteoarthritis, and chronic low back pain [6] and contribute to the impairment of endogenous pain modulation [7] (pain sensitization or pain‐related psychological syndrome) in these individuals.

Exercise has been recommended as a nonpharmacological intervention and overall health promotion for various patients with pain and older adults. The attenuation of chronic pain syndrome and improvement of pain‐related behavior following regular exercise training were known as training‐induced hypoalgesia (TIH), which reflects adaptive changes in pain perception after repeated exercise sessions [8–10]. However, there is no clear evidence proving that the magnitude of the analgesic effects following a single bout of exercise can be improved or restored by regular training in healthy individuals or patients with pain [11].

In healthy individuals, EIH is a multifactorial phenomenon and can be induced by the engagement of descending inhibitory pathways and release of endogenous opioids, endocannabinoids, and monoamines [12–14]. The descending inhibition is modulated by midbrain [15] and cortex [16] when the thalamus [17] receives certain inputs from peripheral nociceptors such as C fibers, involving activation of group III/IV muscle afferents during exercise with sufficient intensity. The EIH magnitude can be affected by conditioned pain modulation (CPM) [18, 19] or temporal summation (TS) [20], which refers to the function of endogenous pain modulation and usually changes in individuals with sensitization of pain perception, which contribute to the impairment of endogenous pain modulation (e.g., altered descending inhibition or facilitation) in these individuals.

Additionally, the EIH magnitude can be modulated by the intensity of exercise [21]; high‐intensity exercises may exacerbate pain in some instances or under certain conditions although they can also induce EIH, suggesting a complex, intensity‐dependent relationship [22, 23], while moderate‐intensity training increases the pain threshold in many conditions [24, 25]. Previous studies [26, 27] have shown that the relationship between EIH and exercise intensity is an inverted U‐shaped curve in healthy individuals. However, the EIH response may be weakened [26] in older adults or patients with painful conditions, and high‐intensity exercise might even exacerbate pain in some cases [28]; the persistent influence on pain perception and endogenous pain modulation may also differ between high‐ and moderate‐intensity exercises.

Therefore, we aimed to compare the persistent effects of high, moderate, and low intensity exercise on EIH and endogenous pain modulation in healthy individuals following 4‐week treadmill running. Given the 4‐week repeated exercise design, this study primarily investigates TIH rather than acute EIH. We measured the effects of EIH in every exercise session and changes in CPM and TS responses before and after the 4‐week training session.

While the relationship between exercise intensity and EIH magnitude may follow an inverted U‐shape curve, it is hypothesized that (1) all intensity levels, including high intensity, can elicit a significant acute EIH response compared to rest, albeit to varying degrees, (2) the magnitude of EIH and CPM responses might gradually improve following regular low and moderate intensity exercise with the attenuation of TS response, and (3) the magnitude of EIH might be correlated with the CPM and TS responses.

## 2. Methods

This study was approved by the Sports Science Experimental Ethics Committee of the Beijing Sport University (approval number: 2023023H).

### 2.1. Study Design

Eighty healthy participants included in this study performed exercise or control interventions 12 times over 4 weeks. All participants provided written informed consent. Demographic data and baseline measurements (resting heart rate (HRrest), PPT, MPT, and CPM responses) were collected. The maximum heart rate (HRmax) was estimated using the following formula [29]: HRmax = 202.5–0.53∗age and the reserved heart rate (HRR) was calculated as HRR = HRmax‐HRrest. Real‐time HR was collected and recorded via the HR belt worn by the participants during running. The first running session was performed 48 h after baseline measurements to avoid potential long‐lasting analgesic effects of the CPM test.

All participants were randomly assigned to three experimental groups (high‐intensity treadmill running [TRH], moderate‐intensity treadmill running [TRM], and low intensity treadmill running [TRL]) and a Control group (keep rest for 30 min). All participants were single‐blinded to treadmill running methods: On the one hand, participants were informed that the running speed was calculated individually and adjusted during running according to their body conditions; on the other hand, participants were not allowed to disclose their own intensity or running speed to others before all running sections were completed. Randomized sequences were generated using Excel software.

**FIGURE 1:**
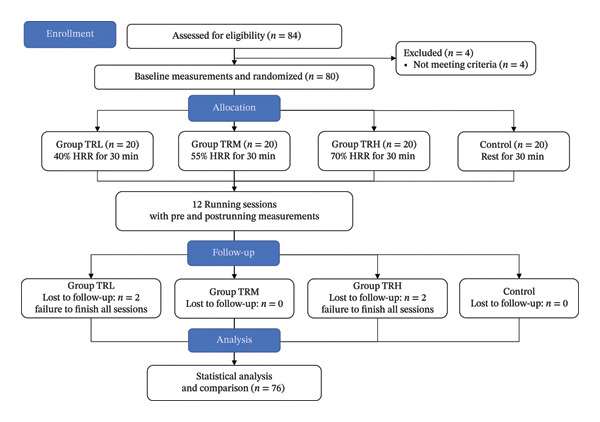
Flowchart of the experiment.

The TRH, TRM, and TRL groups performed treadmill running with 70%, 55%, and 40% HRR for 30 min, respectively. Running speed was determined in accordance with the target heart rate (THR) during baseline measurements. The primary outcomes of this study were PPT and PPT changes before and after exercise sessions (EIH responses). All participants performed a single exercise session once a day, three times per week for 4 weeks (Figure 1).

### 2.2. Participants

Based on previous studies [30], the expected effect size (*f*) for aerobic exercise‐induced PPT changes was 0.38. Our study utilized G∗Power software (Version 3.1) with an effect size *f* = 0.38, an alpha level of 0.05, and a power of 0.80. Thus, a minimum sample size of 48 participants across the three groups was determined.

Eighty‐four healthy students (aged 18–30 years) from Beijing Sports University were assessed for eligibility in this study, of whom 80 were enrolled with 4 excluded.

Exclusion criteria included the following: (1) had any acute or chronic pain condition (e.g., headache, low back pain, and neuropathic pain), or were diagnosed with psychological syndromes such as depression or anxiety disorders within 3 months; (2) had injury history of lower extremities within 1 year; (3) had potential or confirmed heart disease, or recovered from a heart disease < 1 year ago, (4) inability to maintain THR for > 5 min during running; (5) reporting a Borg perceived exertion score > 7 or fatigue visual analog scale (VAS) > 7/10 within 24 h postexercise; (6) reporting pain intensity VAS > 7/10 during any pain test; and (7) using combined hormonal contraceptives or any hormonal contraception (i.e., estradiol and progesterone) which may influence the pain perception. Besides, evidences [31, 32] suggest that the menstrual cycle does not have a significant effect on EIH, so participants who were menstruating would be included in this study.

### 2.3. Procedures

As shown in our previous study [33], all participants completed 12 single‐blinded treadmill running/control sessions over 4 weeks, with intensities tailored to individual THR. Each session comprised three phases: 5 min warm‐up walking at 4 km/h, 30 min running at THR‐maintaining speed (attained via gradual acceleration), and 5 min cool‐down walking at 4 km/h; the control group rested for 30 min instead of running.

Real‐time heart rate (HR) was monitored throughout all sessions using a Polar H10 HR belt (Polar Electro Oy, Finland). One week prior to intervention, all participants underwent a progressive speed‐increment running assessment to determine their THR‐corresponding speed. Treadmill speed was dynamically adjusted during running sessions to sustain THR.

### 2.4. Outcome Measures

The PPT‐arm, PPT‐leg, and MPT were measured 5 min pre and 10 min post each running session. As primary outcomes, PPT‐leg reflected local EIH responses while PPT‐arm represented global EIH responses; MPT was applied to assess sharp pain‐related mechanical perception changes. CPM and TS responses were evaluated pre‐ and postsessions to determine alterations in endogenous pain modulation function. At each testing site, three consecutive PPT/MPT measurements were obtained at 30‐s intervals, with the mean value used for subsequent analysis. Testing order (arm vs. leg) was randomized across participants, and all testing locations were marked with a sterile waterproof marker to ensure repeated‐measure consistency. The algometer was precisely positioned perpendicular to the skin surface during measurements. The reliability of the PPT, MPT, CPM, and TS testing protocols has been investigated in prior studies, with results confirming high inter‐rater and test‐retest reliability [34–38].

### 2.5. PPT (Primary Outcome)

The PPT was assessed via a quantitative sensory testing protocol [39] using a handheld pressure algometer (Baseline Dolorimeter, Fabrication Enterprises, USA) fitted with a 1 cm^2^ metal probe. Pressure was applied at 0.5 kg/s to two right‐sided muscle sites: the extensor carpus radialis (PPT‐arm, 5 cm distal to the lateral epicondyle) and peroneus longus (PPT‐leg, at the muscle belly ∼10 cm distal to the fibular head). PPT was defined as the pressure point at which sensation transitioned to deep pain [40]. Participants were instructed to report “pain” when pressure sensation shifted to definite pain or reached a 30/100 pain intensity (Pain30), and this point was recorded as the PPT value.

### 2.6. MPT

The MPT was measured via a quantitative sensory testing protocol [34] using the same handheld algometer fitted with a needle probe. Pressure was delivered at 0.1 kg/s to the left extensor carpus radialis. Participants rated perceived pain intensity on a 0–100 cm VAS, and the pressure value corresponding to a reported pain intensity of 30/100 (Pain30) was recorded as the MPT.

### 2.7. CPM

The CPM response was assessed via a quantitative sensory testing protocol [39] using the cold pressor procedure. Pressure stimulation at the ipsilateral extensor carpus radialis was used as the test stimulus, with the PPT at Pain30 recorded as the baseline value. Participants then immersed their contralateral hand in 8 °C cold water for 1 min; postimmersion, and PPT at Pain30 was reassessed at the same site. The CPM response was defined as the difference between pre‐ and postimmersion PPT values.

### 2.8. TS

The TS response was measured via a quantitative sensory testing protocol [34]. A needle‐probe algometer was applied to the left extensor carpus radialis at an intensity of 1.25× the participant’s MPT. Ten repeated mechanical stimulations were delivered at 0.5 Hz (1‐s stimulus and 1‐s interval), with timing standardized using a digital metronome. Participants rated pain perception of the first and last stimulations on a 0–100 cm VAS; the TS response was calculated as the difference between these two VAS scores.

### 2.9. Statistical Analysis

The normality of all data was assessed using the Shapiro–Wilk test. Differences in baseline data (height, weight, HRrest, PPT, MPT, CPM, and TS) among groups were compared using one‐way ANOVA. The differences in the PPT and MPT values between pre‐ and postrunning in each session were calculated as EIH responses, including EIH‐A for changes in the PPT‐arm, EIH‐L for changes in the PPT‐leg, and EIH‐M for changes in the MPT.

A two‐way repeated measures ANOVA was chosen as the primary analysis method after confirming that the data met the sphericity assumption and to determine the differences between the three groups over running sessions and examine the EIH values of the PPT and MPT. Also, a one‐way analysis of variance (ANCOVA) was also applied for between‐group comparison of the CPM and TS responses, where the baseline results used as covariates. Post hoc analyses were performed using Bonferroni correction. For all significant effects, both *p* values and effect sizes (*F*‐values) are reported.

The relationships between the CPM and TS, EIH‐A, EIH‐L, and EIH‐M values after the running intervention were analyzed using the Pearson correlation, which was conducted only for variables confirmed to be normally distributed by the Shapiro–Wilk test. All statistical analyses were performed using SPSS Version 21.0, and a significance level of *p* < 0.05 was applied to all tests.

## 3. Results

### 3.1. Baseline Characteristics

Four participants were excluded from this study because of myofascial pain syndrome that occurred 1 month before the experiments. Of the 80 participants enrolled in this study, 18 in the TRL group, 20 in the TRM group, 18 in the TRH group, and 20 in the Control group completed 12 running or control sessions. Additionally, four participants withdrew from the study because of the failure to finish all running sessions. No significant differences were observed in baseline characteristics between the groups (*p* > 0.05, Table 1).

**TABLE 1:** Baseline demographic and pain‐related measurements (M±SD) ^1^.

	TRH (*n* = 18)	TRM (*n* = 20)	TRL (*n* = 18)	Control (*n* = 20)	*p*
PPT‐arm (kg/cm^2^)	2.13 ± 0.30	2.19 ± 0.27	2.06 ± 0.23	2.21 ± 0.30	0.255
PPT‐leg (kg/cm^2^)	3.98 ± 0.48	3.83 ± 0.42	3.96 ± 0.32	3.91 ± 0.34	0.648
MPT (kg/cm^2^)	0.62 ± 0.16	0.57 ± 0.10	0.57 ± 0.07	0.63 ± 0.11	0.109
CPM (kg/cm^2^)	0.58 ± 0.23	0.64 ± 0.30	0.61 ± 0.22	0.62 ± 0.30	0.940
TS (cm)	28.42 ± 6.10	31.99 ± 6.40	27.50 ± 7.67	29.95 ± 6.78	0.194
Age (y)	21.22 ± 1.93	21.30 ± 2.77	21.17 ± 2.04	21.70 ± 2.45	0.891
Height (cm)	169.50 ± 7.61	171.15 ± 7.35	170.33 ± 8.50	171.85 ± 8.94	0.828
Weight (kg)	63.05 ± 12.63	64.75 ± 12.51	65.02 ± 13.04	68.70 ± 13.22	0.576
Male/female	6/12	8/12	7/11	9/11	

*Note:* All data were presented as the mean −/+ standard deviation (M±SD).

^1^: One‐way ANOVA, *p* ≤ 0.05 indicates significance.

### 3.2. Changes in EIH‐A Following Running Sessions

The two‐way repeated measures ANOVA revealed significant effects (*p* < 0.001, *F* = 9.424) for the running sessions involving the EIH‐A, indicating that the 4‐week running intervention significantly increased global EIH responses in all participants. The interaction effect (*p* < 0.001, *F* = 222.498) between running intensities and time for the EIH‐A was also significant. The TRH (*p* < 0.001), TRM (*p* < 0.001), and TRL (*p* < 0.001) groups all presented significant higher EIH‐A increasement compared with the Control group. However, post hoc tests showed that the EIH‐A in the TRM group (*p* < 0.001) since 2^nd^ running session and in the TRL group (*p* < 0.001) since 7^th^ running session were significantly higher than that in the TRH group, which indicated that only the global EIH in the TRM group improved after the 4‐week treadmill running. In addition, all prerunning PPT‐arm values remained unaltered, indicating that regular running may not change the baseline level of the PPT‐arm (Figure 2)).

FIGURE 2Changes in PPT of arms and EIH‐A following running sessions. All data were presented as the mean and standard deviation; PPT = pressure pain threshold; EIH = exercise‐induced hypoalgesia; EIH‐A = EIH value of PPT‐arms; HRR = reserved heart rate; ^∗^: EIH in the TRM group significantly higher than the TRH group; ^#^: EIH in the TRL group significantly higher than the TRH group.

**Figure:**
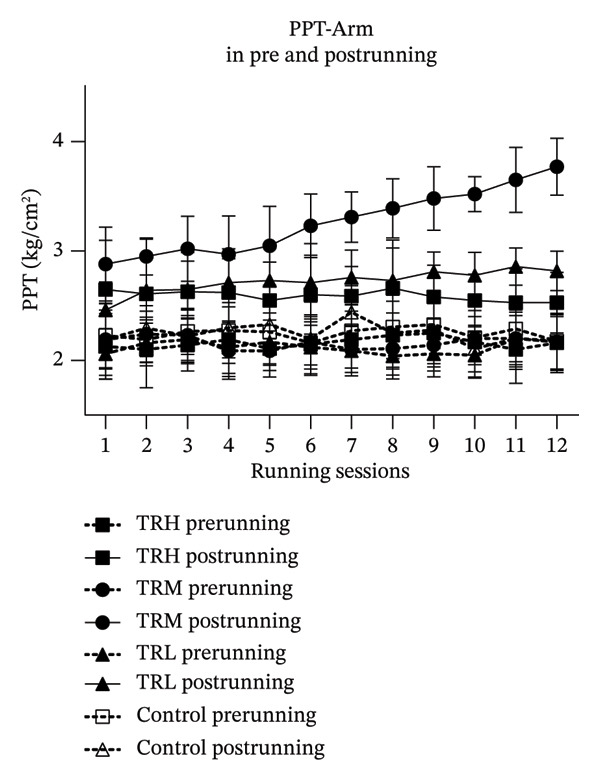
(a)

**Figure:**
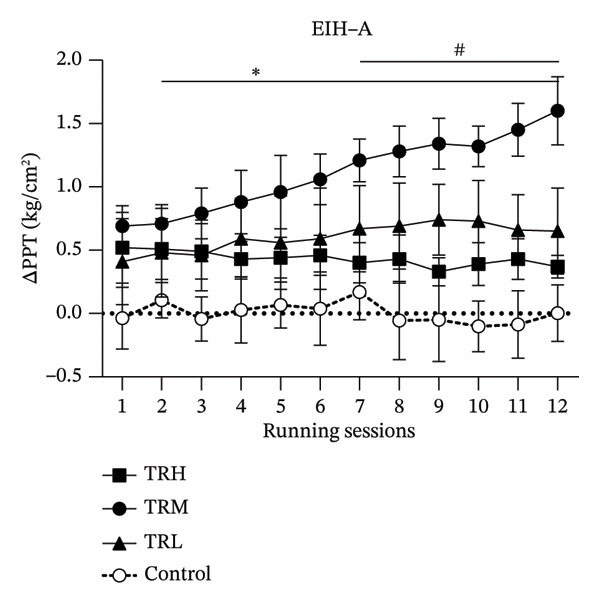
(b)

### 3.3. Changes in EIH‐L Following Running Sessions

Two‐way repeated measures ANOVA revealed significant main effects (*p* < 0.001, *F* = 6.909) for the running sessions involving the EIH‐L, indicating that the 4‐week running intervention significantly increased local EIH responses in all participants. The interaction effect (*p* < 0.001, *F* = 164.129) between running intensities and time for the EIH‐L was also significant. The TRH (*p* < 0.001), TRM (*p* < 0.001), and TRL (*p* < 0.001) groups all presented significant EIH‐L increasement compared with the Control group. However, post hoc tests showed that EIH‐L in the TRM group (*p* < 0.001) since 2^nd^ running session and in the TRL group (*p* < 0.001) since 7^th^ running session were significantly higher (*p* < 0.001) than that in the TRH group, indicating that only the local EIH in the TRM group improved after the 4‐week treadmill running. All prerunning PPT‐leg values remained unaltered, indicating that regular running may not change the baseline level of the PPT‐leg (Figure 3).

FIGURE 3Changes in PPT of legs and EIH‐L following running sessions. All data were presented as the mean and standard deviation; PPT = pressure pain threshold; EIH = exercise‐induced hypoalgesia; EIH‐L = EIH value of PPT‐legs; HRR = reserved heart rate; ^∗^: EIH in the TRM group significantly higher than the TRH group; ^#^: EIH in the TRL group significantly higher than the TRH group.

**Figure:**
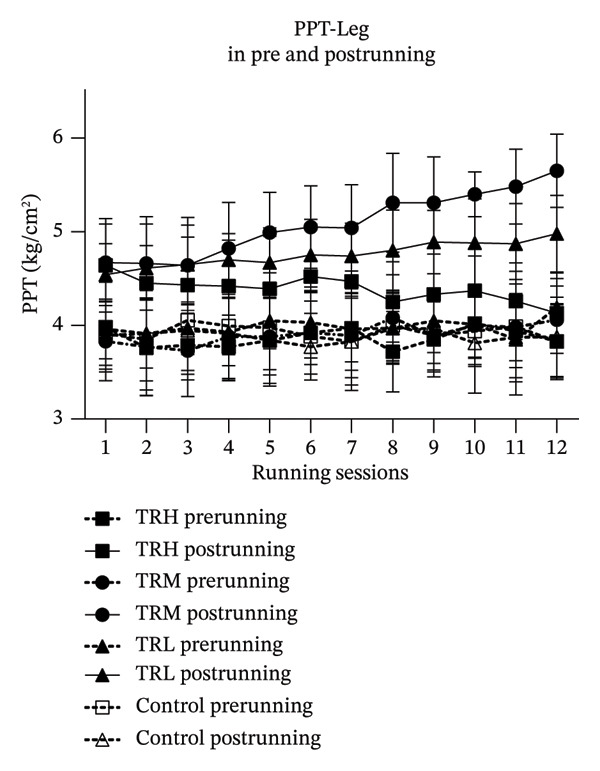
(a)

**Figure:**
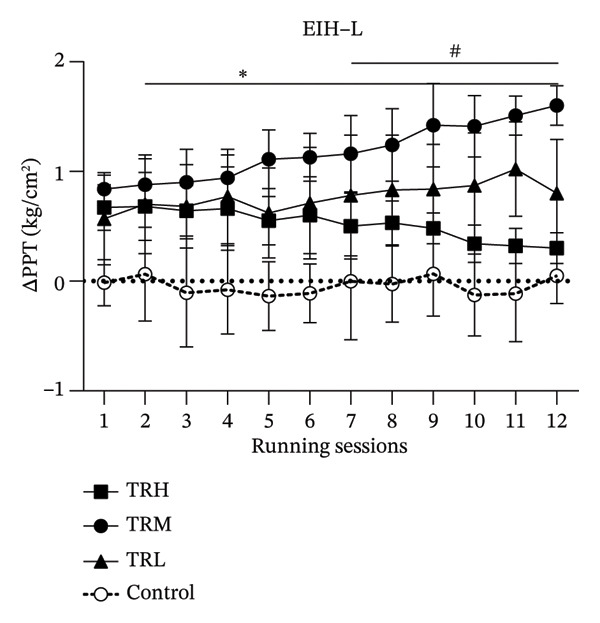
(b)

### 3.4. Changes in EIH‐M Following Running Sessions

The two‐way repeated measures ANOVA revealed significant main effects (*p* = 0.004 and *F* = 2.084) for the running sessions involving the EIH‐M, which indicated that the 4‐week running intervention significantly decreased EIH responses in all participants. And there was no significant interaction effect (*p* = 0.223 and *F* = 1.706) between running intensity and time for the EIH‐M. The TRH (*p* < 0.001), TRM (*p* < 0.001), and TRL (*p* < 0.001) groups all presented significant EIH‐M increasement compared with the Control group. However, the ANCOVA test was applied and showed that the EIH‐M in the TRH group was significantly higher (*p* < 0.001) than that in the TRM and TRL groups in the 1^st^–3^rd^ running sessions. And EIH‐M in the TRM and TRL groups were significantly higher (*p* < 0.001) than that in the TRH group from the 9^th^ to 12^th^ sessions. This result indicated that the EIH‐M in the TRH group gradually decreased following the 4‐week treadmill running, whereas the EIH‐M in the TRM and TRL groups was unaltered during the intervention. Additionally, all prerunning MPT values remained unaltered, indicating that regular running may not change the baseline level of the MPT (Figure 4).

FIGURE 4Changes in MPT and EIH‐M following running sessions. All data were presented as the mean and standard deviation; MPT = mechanical pain threshold; EIH = exercise‐induced hypoalgesia; EIH‐M = EIH value of MPT; HRR = reserved heart rate; ^&^: MPT in the TRH group significantly higher than the TRM and TRL groups; ^∗^: MPT in the TRM group significantly higher than the TRH group; ^#^: MPT in the TRL group significantly higher than the TRH group.

**Figure:**
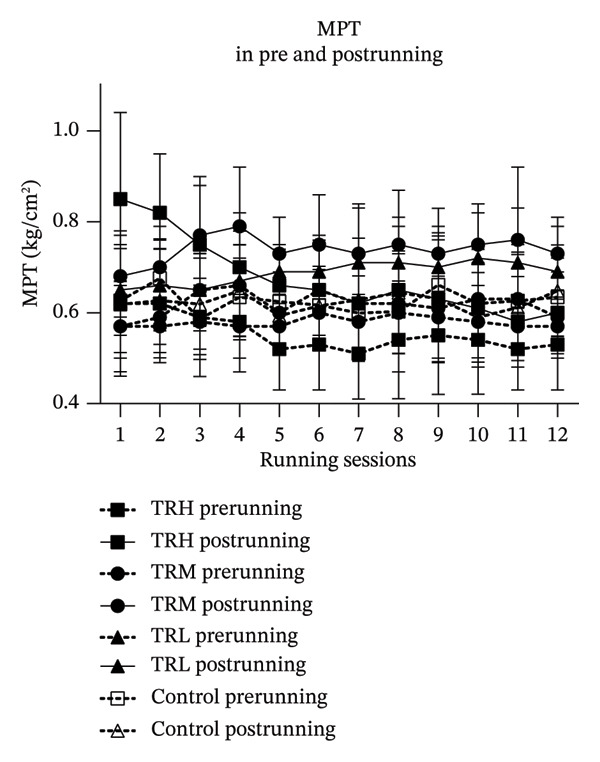
(a)

**Figure:**
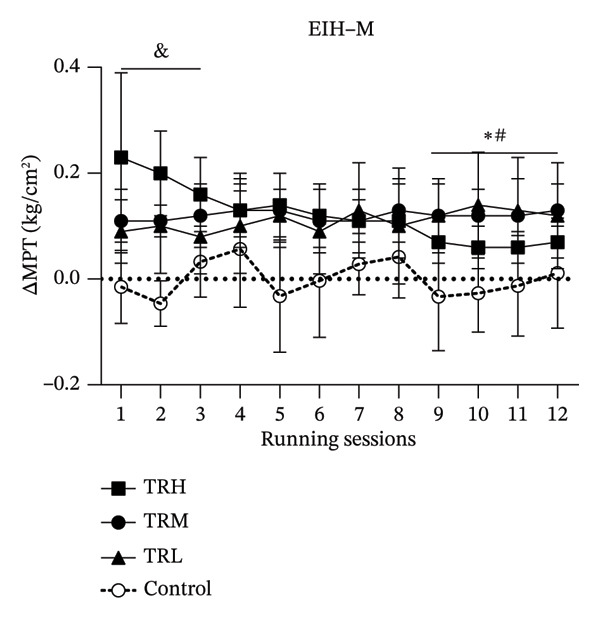
(b)

### 3.5. Changes in CPM and TS Following Running Sessions

One‐way ANCOVA tests revealed significant between‐group differences in the CPM (*p* < 0.001) and TS responses (*p* < 0.001) after the 4‐week treadmill running intervention. The CPM responses in the TRM (*p* < 0.001) and TRL (*p* < 0.001) groups were significantly higher than those in the Control group. In contrast, the TS scores of the TRM (*p* < 0.001) group were significantly lower than those of the Control group, which showed no significant changes before and after the 4‐week running intervention (Figure 5).

FIGURE 5Changes in CPM and TS after 4‐week running. All data were presented as the mean/standard deviation; CPM = conditioned pain modulation; ^∗^: significant difference compared with the TRH group.

**Figure:**
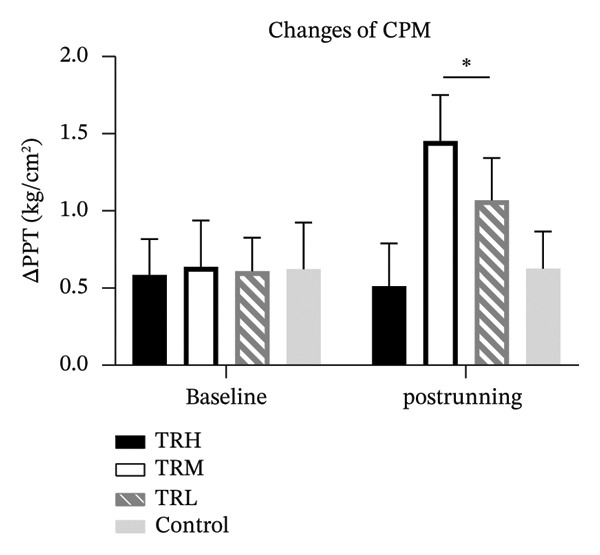
(a)

**Figure:**
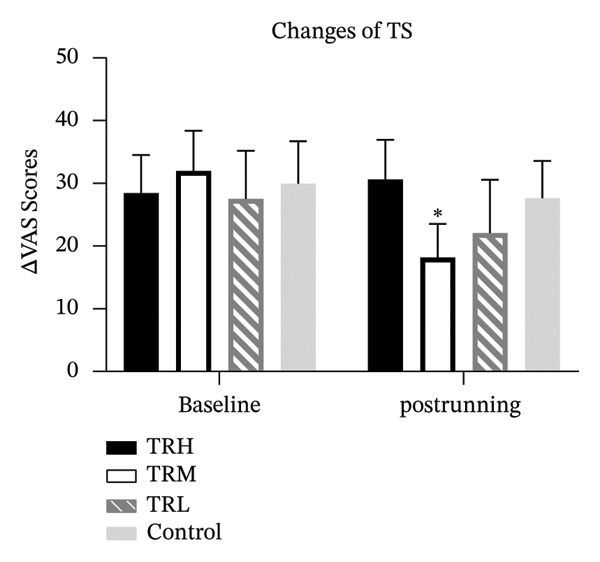
(b)

### 3.6. Relationship Between Endogenous Pain Tests and EIH Magnitudes

**FIGURE 6:**
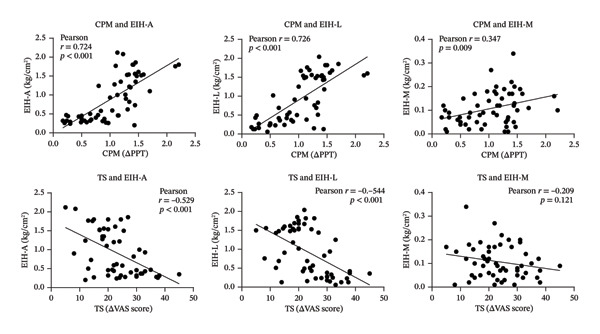
Relationship between CPM/TS and EIH magnitudes; CPM = conditioned pain modulation; TS = temporal summation; EIH‐A = EIH value of PPT‐arms; EIH‐L = EIH value of PPT‐legs; EIH‐M = EIH value of MPT.

Pearson correlation analysis includes all the results in the 12^th^ running sessions and showed that there were significant positive relationships between CPM values and EIH‐A (*r* = 0.724 and *p* < 0.001), EIH‐L (*r* = 0.726 and *p* < 0.001), and EIH‐M (*r* = 0.347, *p* = 0.009) magnitudes. There were also significant negative relationships between TS values and EIH‐A (*r* = −0.529 and *p* < 0.001) and EIH‐L (*r* = ‐0.544 and *p* < 0.001) magnitudes. However, the EIH‐M (*r* = ‐0.209 and *p* = 0.121) magnitudes showed no significant relations to TS scores (Figure 6).

## 4. Discussion

We aimed to investigate changes in pain perception, EIH, and endogenous pain modulation responses following 4‐week treadmill running exercises of different intensities in healthy individuals. Our results revealed the following: First, low‐, moderate‐ and high‐intensity treadmill running may induce the acute analgesic effects, with improvements in global and local PPT and MPT. Second, acute EIH responses following a running session varied according to the type of pain perception and exercise intensity, where the EIH‐A and EIH‐L following low and moderate intensity running were significantly increased along with exercise sessions, and the EIH‐A and EIH‐L following high intensity running slightly decreased after the intervention. Third, the EIH‐M following high intensity running significantly decreased with exercise sessions, and the EIH‐M following low and moderate intensity running remained unaltered. Finally, the CPM was enhanced, and TS was decreased following the moderate intensity exercise, showing positive and negative correlations with EIH responses, respectively. The observed improvements in EIH and CPM following 4 weeks of training suggest a cumulative adaptation (TIH), rather than merely an acute session effect.

The baseline pain perception threshold is relatively constant in healthy individuals during 4‐week running [41] and may not be affected by short‐term regular exercise training. Recent studies have shown that a 24‐week high‐intensity interval training [42] and a 20‐week resistant band exercise [43] have no significant effects on the PPT in healthy individuals. Tesarz et al. [44] investigated baseline pain perceptions in athletes and normally active individuals and observed that differences in pain thresholds between the groups were not significant.

However, as a response to endogenous pain modulation, the acute EIH in PPT changes following exercise may improve after a regular exercise intervention. Song et al. [11] suggested that exercise training induces physiological changes leading to improved EIH. Ohlman et al. [5] observed a greater EIH response in individuals who performed moderate physical activity per week than in sedentary controls. Hansen et al. [45] also observed that the PPT and EIH in healthy individuals significantly increased after a 7‐week military training. However, evidence from randomized trials with a pre‐test–post‐test design remains limited.

Exercise with a sufficient load can induce a short‐term EIH response, whereas exercise with low‐to‐moderate intensity may elicit greater analgesic effects in PPT than high‐intensity or exhaustive exercise [26, 27]. Meanwhile, it is plausible that running at moderate intensity may preferentially activate non‐noxious afferent C fibers [46] via repeated muscle contractions and engage descending inhibitory pathways, potentially involving serotonergic mechanisms in the brainstem [47], which could contribute to the improvement of PPT, EIH‐A, and EIH‐L than low‐intensity exercise.

Additionally, the CPM positively correlated with EIH‐A and EIH‐L, indicating that the improvement of EIH may have been induced via the changes of descending inhibition. While the TS negatively correlated with EIH in PPT, it showed the potential pain prevention effects of regular running. Lemley et al. [18] investigated EIH in healthy individuals and observed that those with greater CPM were more likely to experience greater EIH. Naugle et al. [48] also observed that healthy adults who self‐reported increased total physical activity exhibited reduced TS and greater CPM. Thus, the low or moderate intensity exercise may enhance the central descending inhibition function and increase acute EIH responses in PPT with an increase in CPM responses.

High‐intensity exercise may trigger noxious [40] and non‐noxious C fibers and potentially induce descending facilitation [41] with limited EIH‐A and EIH‐L responses. Meanwhile, the upregulation of cannabinoids and opioids expression following high‐intensity exercise may decrease the perception of mechanical stimuli and evoke acute EIH‐M effects [42].

However, the responsivity of opioids system may reduce following repeated high‐intensity exercise stimulation and then lower the EIH‐M volume. For example, athletes experiencing high‐intensity training showed a partially decreased EIH response than did healthy controls. Siebers et al. [49] observed a downregulation of endocannabinoid levels following regular running training. Therefore, repeated high‐intensity exercise may lead to adaptations in the endogenous cannabinoid and opioid systems, which could potentially account for the observed reduction in mechanical analgesic effects over time.

### 4.1. Limitations

Our study had several limitations. First, the indicators of the pain tests were limited. For instance, adding heat pain detection thresholds might provide a more complete description of the changes in pain perception, and the lack of blinding of outcome assessors may also have introduced potential bias. Second, the intervention period for running exercise was relatively short. Future studies should investigate the long‐term (> 8–12 weeks) effects on pain perception and modulation following various types of exercises and also include nonexercise control group. Third, the ipsilateral CPM protocol was chosen to minimize contralateral interactions and better reflect pain modulation. However, the use of a fixed 8°C for 1 min may not be sufficient to induce robust pain inhibition in all participants, which is a limitation of the current protocol. Fourth, the male/female ratios in groups were not balanced. However, previous studies have found that sex hormones might affect pain perception and analgesic mechanisms. Additionally, although current evidence [31] suggests that the menstrual cycle may not significantly affect EIH, we did not control for menstrual phase in female participants, which could potentially influence pain perception. Finally, participants consisted only healthy young adults, which limits the generalizability of the findings to older individuals, clinical populations, or those with different fitness levels.

Compared with studies in chronic pain populations (e.g., fibromyalgia and chronic low back pain), where EIH and CPM are often impaired, our findings in healthy adults suggest that regular exercise may serve as a preventive strategy to maintain or enhance endogenous pain modulation. Future research should further optimize the gender ratio and clinical populations with impaired pain modulation, such as fibromyalgia or chronic low back pain, and comprehensively assess the analgesic effects of exercise and its mechanisms.

## 5. Conclusion

In summary, both moderate‐ and low‐intensity treadmill running improved acute EIH responses compared with high‐intensity running in healthy individuals. CPM and TS may be correlated with EIH and changed after regular exercise, indicating that treadmill running may improve EIH through functional changes in endogenous pain modulation.

## Author Contributions

Zihan Xu, Nan An, Shuang Xu, and Yue Li conceived and designed the research; Zi‐Han Xu, Nan An, Ruyun Wang, and Yue Li performed experiments; Zihan Xu, Shuang Xu, and Yue Li analyzed data. Zihan Xu, Nan An, Shuang Xu, Ruyun Wang, and Yue Li interpreted results of experiments. Zihan Xu and Yue Li prepared Tables and Figures and drafted the manuscript. All authors edited and revised the manuscript drafts.

## Funding

No funding was received for this manuscript.

## Disclosure

This article has a preprint at https://www.medrxiv.org/content/10.1101/2024.03.27.24304823v4 [33]. We confirm that the results of the study are presented clearly, honestly, and without fabrication, falsification, or inappropriate data manipulation. We also confirm that the results of the present study do not constitute endorsement by ACSM. All authors approved the final manuscript.

## Conflicts of Interest

The authors declare no conflicts of interest.

## Data Availability

The data that support the findings of this study are available from the corresponding author upon reasonable request.
